# Fiat Justitia

**Published:** 1890-06

**Authors:** 


FIAT JUSTITIA.—In our last issue appeared a letter from Dr. Leake, submitted to the JOURNAL for an opinion as to the use of certain words. In the editorial comments there on, Dr. Leake feels that injustice was done him, and that said remarks detracted from the value of certain published papers. It affords us pleasure to say that the Doctor has both misapprehended our meaning and our animus; and that nothing was further from our thoughts than either doing him injustice, reflecting on him, or detracting from the merits of his papers. The word "hypocritical" to which reference was made was, so we are informed, a clerical error; "hypercritical" being the word intended, and dictated to his typewriter. For the Doctor we have the highest regard, both as a gentleman and a physician, and it gives us pleasure to "set him right," before the profession

